# The importance of lipids for neurodevelopment in low and middle income countries

**DOI:** 10.3389/fnut.2025.1488647

**Published:** 2025-06-24

**Authors:** Pervy Okai-Mensah, Diandra Brkić, Jonas Hauser

**Affiliations:** Nestlé Institute of Health Sciences, Nestlé Research, Société des Produits Nestlé S. A., Lausanne, Vaud, Switzerland

**Keywords:** fatty acids, neurodevelopment, low and middle-income countries, early life, pregnancy

## Abstract

This review focuses on the effects of polyunsaturated fatty acids (FA) supplementation on neurodevelopmental outcomes in the first year of life in low- and middle-income countries (LMIC). Lipids are an essential part of early life diet; they provide crucial FAs for brain development and healthy growth. The high cost of relevant food sources providing specific FAs restricts their use and consumption in LMIC where more than 3 billion people cannot afford a healthy diet. This narrative review summarizes current knowledge extracted from 24 studies on the impact of specific FAs on neurodevelopment from birth to 12 years of age, with a particular focus on LMICs. We illustrate that most studies focus on effects of polyunsaturated FAs supplementation on neurodevelopmental outcomes in the first year of life. The strongest evidence in the literature is on supplementation during pregnancy with omega-3 fatty acids, in particular alpha-linolenic acid (ALA) and omega-6 fatty acids, in particular linoleic acid (LA), which show promising effects on infant neurodevelopmental outcomes in LMIC. These two essential fatty acids (EFAs) are key substrates necessary to synthesize the long-chain poly-unsaturated fatty acids (LC-PUFA) docosahexaenoic acid (DHA) and arachidonic acid (ARA), which have been reported to be important for neurodevelopment. For the postnatal supplementation we did not observe a clear consensus across studies, either due to mixed finding before 2 years of life or due to the low number of studies beyond 2 years of life. Differences across studies in the choice of FAs, dosage, treatment windows, age and type of neurodevelopment assessments likely contribute to the complexity of the results observed in the studies investigating postnatal supplementation. Finally, this review underlies the need for more research into FAs that support optimal development of children in LMICs and highlight the importance to find affordable solutions without compromising on quality.

## 1 Introduction

A recent report by Black et al. (1) shows that 250 million children living in low- and middle-income countries (LMICs), fail to reach their optimal developmental potential due to poverty and malnutrition (2). This is concerning given that adequate nutrition has been recognized as one of the fundamental pillars of optimal development (3). Too many children in LMICs suffer from poor diet diversity and quality due to economic constraints. Early life malnutrition has long-lasting effects on cognitive and physical development, which in turn affects later success in life, such as academic performance and employment opportunities (4–7). A healthy diet, especially in early years, is crucial for optimal neurodevelopment and benefits society at large.

Malnutrition is a major risk factor for suboptimal neurodevelopment because dietary nutrients provide structural building blocks and/or modulators of various neurodevelopmental processes such as neurogenesis, neuronal differentiation, myelination and synaptogenesis, all of which peak during early life. Some non-exhaustive examples of key nutrients important for neurodevelopment are docosahexaenoic acid (DHA), vitamin A, B-vitamins, copper, iron, iodine, and zinc. Multiple non-profit organizations, including World Health Organization (WHO), have reported that lower socioeconomic status (SES) has been associated with poor diet and the consequent negative impact on neurodevelopment (8–10) highlighting the importance for the development of a more affordable and healthy diet (11, 12). Recent leverage of Magnetic Resonance Imaging (MRI) technology even demonstrated that specific fatty acids supplementation resulted in change of brain size in newborn, highlighting that maternal diet can impact brain growth (13). Currently, the WHO defines a healthy diet as a diet that helps protect against malnutrition in all its forms as well as non-communicable diseases, but healthy diets are not accessible by many low SES populations. For these groups, the costs of a healthy diet are higher than the estimated purchase power, computed as 62% of the 1.9 United States $ per day income that can be devoted to food.

The majority of studies investigating the role of nutrition in neurodevelopment in LMICs has focused on micronutrients, namely vitamins and minerals (14–18), while research on other dietary components, such as fatty acids (FAs), is still limited. This is surprising given the rising interest in the benefits of essential FAs (EFAs), such as alpha-linolenic acid (ALA), an omega-3 FA and linoleic acid (LA), an omega-6 FA, and (LC-PUFA), such as docosahexaenoic acid (DHA) and arachidonic acid (ARA), on neurodevelopment.

Lipids represent 50%–60% of the human brain’s dry weight (19, 20). Of this, 25% is composed of LC-PUFAs DHA (an omega-3 FA) and ARA (an omega-6 FA), two core FAs concentrated in gray matter (19, 20). LC-PUFAs cannot be formed *de novo* but are synthesized from parent EFAs, LA, and ALA, primarily coming from the diet (21–23). The primary source of EFAs for infants comes from the mother, via the placenta before and via breast milk after birth (24). With the introduction of solid foods in the diet, the sources of EFA include plant oils, such as soybean, flaxseed, and canola oils as well as fish (i.e., salmon, mackerel, tuna, herring, and sardines), fish oils, and krill oils. In addition to their role as basic components of neuronal membranes, EFAs have various modulatory roles on the developing central nervous system. More specifically, EFAs modulate membrane fluidity and volume by impacting receptor and enzyme activities as well as ion channels. Moreover EFAs can be used as precursors for active mediators, like eicosanoids and prostaglandins, that play a key role in inflammation and immune reactions. EFAs also have an important role in neural signal processing and transmission by promoting neuronal and dendritic spine growth and synaptic membrane synthesis. Furthermore, EFAs regulate gene expression in the brain (22, 25–32). Given the copious amount of evidence and studies investigating brain benefits, EFAs are critical for neurodevelopment.

Beyond EFAs supplementation, several studies investigated the potential health benefits of LC-PUFAs, showing that high intake levels of DHA in pre- and postnatal periods improve cognitive skills ranging from processing ability, attention, to overall IQ, with benefits noticeable up to 12 years of age (33–35). Similarly, DHA insufficiency has been proposed to have preclinically and clinically negative impact on brain development (36). A growing body of evidence has also identified more complex lipids such as polar lipids (phospholipids and sphingolipids) supporting neurodevelopment through regulation of signal transduction, myelination, and synaptic plasticity (37).

However, there is a paucity of research investigating the role of complex lipids in neurodevelopment specifically in LMIC. Therefore, the first objective of this narrative review is to gather current knowledge on the impact of dietary lipids, focusing on EFAs and LC-PUFAs, on brain development and cognition in children from birth to 12 years old living in LMICs. Second, we discuss the importance of affordable solutions for LMIC.

## 2 Methods

### 2.1 Literature search

Literature searches were performed between March 2022 and April 2025, in PubMed and Google Scholar, for publications between 2000 and 2024. These two databases were selected to ensure we covered most literature in the field. Study risk of bias was individually evaluated and judged to be small for all studies. We did not use any statistical approach due to the technical differences between all the studies [duration of treatment, composition of treatment, time of readout, type of study (RCT vs. observational)]. The following keywords related to lipids and neurodevelopment in the LMIC context were included: affordable, cost, lipids, fatty acids, preconception, maternal, school-age children, children, pregnancy, prenatal, infants, toddlers, adolescents, young adults, neonatal, conception, teen, cognition, school-performance, attention, learning, memory, language (expressive, receptive), motor function, sleep, socio-emotional function, temperament, executive function, decision making, working memory, long-term memory, sleep, communication, brain malformation, cognitive deficits, motivation, resilience, willpower, perception, vision, visual acuity, self-regulation, focus, concentration, recall, short term memory, academic performance, assessment, cognitive ability, long-term storage/retrieval, visuo-spatial ability, intelligence, intellectual functioning, early childhood development, brain volume, white matter volume, white matter integrity, gray mater volume, gray matter integrity, connectivity, network function, electroencephalography (EEG), brain metabolism, Magnetic Resonance Imaging (MRI), Magnetic Resonance Spectroscopy (MRS), Diffusion Tensor Imaging (DTI), Functional Near-infrared spectroscopy (FNIRS), morphology, myelination, synaptogenesis, brain development, brain mass, neurons, cerebral blood flow, neurogenesis, malnutrition, deficiency, child nutrition, diet, supplementation, food, nutritional requirements, dietary intake, socio-economic status, low and middle income countries, poverty, developing countries, Africa, Asia, South America, Eastern Europe.

### 2.2 Inclusion and exclusion criteria

Eligible studies had the following inclusion criteria: (1) studies done in LMIC as defined by World Bank income categories in 2019–2020 (38); (2) studies including pregnant women, breastfeeding mothers and children from birth to 12 years of age; (3) all types of studies were eligible, i.e., randomized clinical trials (supplements, staple foods, or lipid enriched foods interventions), and observational studies.

Exclusion criteria were the following: (1) subjects affected by a physical, neurological, or psychological disorder, suffering from malnourishment or growth faltering; (2) studies investigating nutritional intervention not composed of lipids; (3) studies not investigating cognition.

### 2.3 Neurodevelopmental outcomes selection

Neurodevelopmental outcomes assessed included neurodevelopment/neural development, motor development, language development (expressive and receptive), executive function(s), socio emotional development, brainstem auditory evoked potentials, visual development, coordination and acuity, cerebral blood flow, working memory, short term memory, fluid reasoning, and plasma fatty acid status. Outcomes could be measured at any stage, from prenatal to school age (up to 12 years).

## 3 Results

Hundred and fifty-three (*N* = 153) publications were identified with the search strategy, of which 25 were selected after applying the inclusion/exclusion criteria (see Supplementary Table 1). Of the 25 studies selected, thirteen were conducted in Africa, six in Asia, and five in Central America and one in Europe. Countries included were India, Guinea Bissau, Ghana, Tanzania, Malawi, Mexico, Cuba, Gambia, Bangladesh, Pakistan, Turkey, South Africa and Seychelles. Twelve of the studies focused on the prenatal stage (see Table 1), 11 on infants (0–2 years, see Table 2), and five on preschooler and school aged children (3–12 years see Table 3), with these numbers not mutually exclusive as some studies covered more than one of these age ranges.

**TABLE 1:** Summary of randomized controlled trial intervention that used supplementation during pregnancy.

References	Type of study	Fatty acid intervention/assessment	Type of lipids in intervention or analyses	Country	Neurodevelopmental assessment tools	Results
**Interventions**						
Khandelwal et al. (46)	RTC with intervention	400 mg/d algal DHA from at most from 20 weeks pregnancy till 6 m postpartum	LC-PUFA	India	Development Assessment Scale for Indian Infants to Assess Neurodevelopment^#^	Null findings - no effect on neurodevelopment at 12 months of age
Matias et al. (39)	RTC with intervention	LA (4.59 g) and ALA (0.59 g) to mothers > 20 weeks pregnancy – 6 months. Postpartum. LA (4.46 g) and ALA (0.58 g) for infants 6–24 months (including multi vitamins and multi minerals)	EFA	Bangladesh	Developmental Milestones Checklist II*; Macarthur-Bates Communicative Development Inventory for expressive and receptive language*.	Positive findings - improved motor performance and receptive language at 18 months of age but not in expressive language. Later at 24 months, lipid supplemented children performed much better in expressive language.
Ocansey et al. (40)	RTC with intervention	LA (4.59 g) and ALA (0.59 g) to mothers > 20 weeks–6 months postpartum. LA (4.46 g) and ALA (0.58 g) for infants 6–18 months (including multi vitamins and multi minerals)	EFA	Ghana	Strengths and Difficulties Questionnaire*; Developmental Neuropsychological Assessment II^#^; block design^#^; visual search^#^; delayed gratification^#^; memory task^#^; head-toe test^#^; NIH toolbox pegboard test for fine motor function^#^.	Positive findings - decreased behavioral problems but no effect on cognition, gross and fine motor functioning, socio-emotional regulation at 4–6 years old.
Prado et al. (45)	RTC with intervention	LA (4.59 g) and ALA (0.59 g) to mothers from at most 20 weeks till 6 months postpartum (including multi vitamins and multi minerals)	EFA	Ghana	Kilifi Developmental Inventory (KDI) for motor development^#^	Positive findings - improved motor development at 12 months but not at 18 months.
Ramakrishnan et al. (49)	RTC with intervention	400 mg/day of DHA starting from 18 to 22 weeks of gestation through delivery	LC-PUFA	Mexico	Bayley Scales of Infant Development-II (BSID II)^#^	Null findings - no effect on mental and psychomotor development at 18 months.
Ramakrishnan et al. (51)	RTC with intervention	400 mg of DHA starting from 18 to 22 weeks of pregnancy until delivery.	LC-PUFA	Mexico	Conners’ Kiddie Continuous Performance Test for attention^#^	Positive findings - increased sustained attention in children at 5 years.
Stein et al. (50)	RTC with intervention	400 mg of DHA starting from 18 to 22 weeks of pregnancy until delivery.	LC-PUFA	Mexico	Sierra wave instrument (Cadwell laboratories) for auditory and visual evoked potentials^#^	Null findings - no effect on brainstem auditory-evoked responses at 1 and 3 months or visual-evoked potentials at 3 and 6 months.
Tofail et al. (48)	RTC with intervention	Fish oil, DHA (1.2 g), EPA (1.8 g) in intervention and soy-oil containing 2.25 g LA and 0.27 g ALA > 25 weeks of pregnancy till delivery	LC-PUFA	Bangladesh	Bayley Scales of Infant Development-II (BSID II)^#^	Null findings - no effect on mental and psychomotor development at 18 months.
Aryee et al. (43)	RCT with intervention	Small quantity lipid-based nutrient containing LA (4.59 g or ALA 0.59 g)	EFA	Ghana	Autonomic nervous system assessed with Emotion Go/No-Go (EGEG) and RACER Simon^#^	Null findings - no impact on impact on autonomic nervous system.
Imtiaz et al. (44)	RCT with intervention	Lipid based nutrient supplement containing LA (4.59 g or ALA 0.59 g) with or without health education from < 20 weeks of pregnancy until 23 mo of age	EFA	Pakistan	Caregiver-Reported Early Development Instrument (CREDI)*	Positive findings - supplementation and education resulted in positive effect on cognition, motor and language development at 2 years and in those as well as social-emotional function at 32 months of age.
**Observations**						
Parra-Cabrera et al. (41)	Observational	Evaluation of maternal dietary intake for PUFA, with specific focus on DHA and ARA	LC-PUFA	Mexico	Brainstem auditory evoked potential (BAEP)^#^	Positive findings - higher BAEP were associated with ARA, but not DHA in infants from 3 to 12 months.
Strain et al. (47)	Observational	Measurement of maternal EFA and PUFA status, but reported only DHA and ARA or total omega-3 and total omega-6 at 28 weeks gestation	LC-PUFA	Seychelles	Clinical Evaluation of Language Fundamentals^#^; Kaufman Brief Intelligence Test^#^; Woodcock-Johnson Test of Achievement–III^#^; Boston Naming Test ^#^and Trailmaking^#^ for EF.	Null findings - No association between maternal DHA and ARA status with language, cognition, scholastic achievement and executive function at age 7 years.

RCT, randomized control trial; ALA, alpha-linolenic acid; LA, linoleic acid; EPA, eicosapentaenoic acid; DHA, docosahexaenoic acid; ARA, arachidonic acid; KDI, Kilifi Developmental Inventory; K-CPT, Conners’ Kiddie Continuous Performance Test; EFs, executive functions; BSID, Bayley Scales of Infant Development; BAEP, brainstem auditory evoked potential. All studies included were focusing on healthy subjects, as one of the exclusion criteria of this review was presence of physical, neurological, or psychological disorder, suffering from malnourishment or growth faltering.

* indicates test that consists of questionnaires to the parents or caregivers and ^#^ indicates assessment of the subjects.

**TABLE 2:** Summary of the studies that focused on effects of lipid supplementation effects from infancy to 24 months of age.

References	Type of study	Fatty acid intervention/assessment	Type of lipids in intervention or analyses	Country	Neurodevelopmental assessment tools	Results
**Interventions**						
Matias et al. (39)	RCT with intervention	LA (4.59 g) and ALA (0.59 g) to mothers > 20 weeks pregnancy – 6 months. postpartum; LA (4.46 g) and ALA (0.58 g) for infants 6–24 months (including multi vitamins and multi minerals)	EFA	Bangladesh	Macarthur-Bates Communicative scale for language*; A-not-B-task^#^ for EF; Developmental Milestones checklist-II* for motor development	Positive findings - lipid group children improved motor performance and receptive language at 18 months. Lipid group at 24 months. did better at expressive language. No difference in EF.
Ocansey et al. (40)	RCT with intervention	LA (4.59 g) and ALA (0.59 g) to mothers > 20 weeks–6 months postpartum. LA (4.46 g) and ALA (0.58 g) for infants 6–18 months (including multi vitamins and multi minerals)	EFA	Ghana	Developmental Neuropsychological assessment II – language^#^; Parental questionnaire for developmental status and milestones*; head-toe-knee-shoulders delayed gratification^#^ for EF; visual search tasks^#^, British Ability Scale II (BAS)^ #^ and Wechsler primary and preschool for Intelligence^#^ for IQ and visuospatial ability; Baddley memory task^#^ for WM; NIH toolbox^#^ for motor function; Strength and difficulties questionnaire* for socio-emotional questionnaire.	Positive findings - decreased behavioral problems reported by caregivers at preschool age, especially for low stimulation households, but no effects on cognition or motor function at 4–6 years old (preschool age).
Phuka et al. (54)	RCT with intervention	LA (3.6 g) and ALA (0.4 g), or LA (7.3 g) and ALA (0.8 g) to 6 months old infants for 12 months (including multi vitamins and multi minerals)	EFA	Malawi	Griffiths’ Developmental Assessment Tool^#^ for cognitive development.	Null findings - no effect on cognitive development at 18 months.
Prado et al. (42)	RCT with intervention	LA (4.59 g) and ALA (0.59 g) to mothers > 20 weeks until 6 months. postpartum; LA (4.46 g) and ALA (0.58 g) to offspring 6–18 months (including multi vitamins and multi minerals)	EFA	Ghana	Kilifi Developmental Inventory^#^ for motor development; Macarthur-Bates Communicative Development Inventory* for language; Profile of social and emotional development*; A-not-B-task^#^ for EF	Positive findings - improved motor development at 12 months, but not at 18 months. Null findings - no difference in EF, motor, language or socio-emotional functions at 18 months.
Prado et al. (45)	RCT with intervention	LA (4.59 g) and ALA (0.59 g) to mothers > 20 weeks until 6 months. postpartum; LA (4.46 g) and ALA (0.58 g) to offspring 6–18 months (including multi vitamins and multi minerals)	EFA	Malawi	KDI^#^ for motor development; Macarthur-Bates Communicative Development Inventory* for language; Profile of social and emotional development*; A-not-B-task^#^ for EF	Null findings - Lipid based supplements taken from 6 to 18 months had no effect on motor, language, EF, and socioemotional development at 18 months.
Roberts et al. (53)	RCT with intervention	DHA (255 mg) and EPA (171 mg) for children 15 months–7 years old for 23 weeks (including multi vitamins and multi minerals)	LC-PUFA	Guinea Bissau	Spin the pots task^#^ for WM; NIRS-DCS^#^ for cerebral flow.	Positive findings - increased cerebral blood flow and better WM was observed in children.
Unay et al. (52)	RCT with intervention	DHA supplemented formula (0.5/100 g fat) to infants from 0 to 16 weeks (duration = 16 weeks).	LC-PUFA	Turkey	Brainstem auditory evoked potentials^#^	Positive findings - infants fed on human milk or a formula supplemented with LCPUFAs during the first 16 weeks showed more rapid brainstem auditory evoked potential maturation vs. standard formula-fed infants.
Van der Merwe et al. (55)	RCT with intervention	Fish oil supplementation with DHA (200 mg) and EPA (300 mg) for 3–9 m infants for 12 months	LC-PUFA	Gambia	Willatts’ 2-Step Infant Planning Test^#^ for EF	Null findings - supplementation successfully increased plasma n-3 FA status in infants but had no effect on cognitive development post 6 months. of intake.
Aryee et al. (43)	RCT with intervention	Small quantity lipid-based nutrient containing LA (4.5 g or ALA 0.6 g) from 6 months of pregnancy until 18 months of age	EFA	Ghana	Autonomic nervous system assessed with Emotion Go/No-Go (EGEG) and RACER Simon^#^	Null findings - no impact on impact on autonomic nervous system.
Imtiaz et al. (44)	RCT with intervention	Lipid based nutrient supplement containing LA (4.5 g or ALA 0.6 g) with or without health education from < 20 weeks of pregnancy until 23 mo of age	EFA	Pakistan	Caregiver-Reported Early Development Instrument (CREDI)*	Positive findings - supplementation and education resulted in positive effect on cognition, motor and language development at 2 years and in those as well as social-emotional function at 32 months of age.
**Observations**						
Krasevec et al. (56)	Observational	Evaluation of breast milk and plasma 2 months postpartum to assess FA profiles of total lipids (DHA)	EFA and LC-PUFA	Cuba	Teller acuity cards^#^ to measure visual acuity.	Null findings - no association between maternal DHA and infant visual acuity at 2 months of age.

RCT, randomized control trial; ALA, alpha-linolenic acid; LA, linoleic acid; EPA, eicosapentaenoic acid; DHA, docosahexaenoic acid; FA, fatty acids; EFs, executive functions; BAS, British Ability Scale II; WM, working memory; NIRS, near infrared spectroscopy; DCS, diffuse correlation spectroscopy; LCPUFA, long-chain polyunsaturated fatty acids. All studies included were focusing on healthy subjects, as one of the exclusion criteria of this review was presence of physical, neurological, or psychological disorder, suffering from malnourishment or growth faltering.

* indicates test that consists of questionnaires to the parents or caregivers and ^#^ indicates assessment of the subjects.

**TABLE 3:** Summary of the studies that focused on effects of lipid supplementation effects from 2 years and above.

References	Type of study	Fatty acid intervention/assessment	Type of lipids in intervention or analyses	Country	Neurodevelopmental assessment tools	Results
**Interventions**						
Dalton et al. (58)	RCT with intervention	ALA (335.02 mg), EPA (82.16 mg), DHA (191.66 mg), LA (1567.36 mg), ARA (23.25 mg) in a fish flour spread in children 7–9 years old for 6 months.	EFA and LC-PUFA	South Africa	Hopkins Verbal Learning Test^#^ for cognitive functioning, Reading and Spelling^#^ was also assessed.	Positive findings - learning ability and memory improved after implementation containing LCPUFA
Muthayya et al. (60)	RCT with intervention	ALA (930 mg), DHA (100 mg), or ALA (140 mg) for 12 months in children 6–10 years old (including multi vitamins and multi minerals)	EFA and LC-PUFA	India	Kaufman Assessment Battery for Children^#^, WISC^#^; Rey Auditory Verbal Learning test^#^; and Neuropsychological assessment tool^#^ for short term memory, fluid reasoning, cognitive Speediness and overall cognition.	Null findings - FA supplementation made no difference among the groups on short term memory, fluid reasoning, cognitive speediness and overall cognition.
Roberts et al. (53)	RCT with intervention	DHA (255 mg) and EPA (171 mg) in children from 15 months to 7 years old for 23 weeks (including multi vitamins and multi minerals)	LC-PUFA	Guinea Bissau	Spin the pot task (variation)^#^ for WM; NIRS^#^ and DCS^#^ for cerebral flood.	Positive findings - increased cerebral activity (blood flow) and better WM were observed in children < 4 years old but not in the four and above.
**Observations**						
Adjepong et al. (57)	Observational	analysis whole-blood FA profile in 2–6 years olds	EFA and LC-PUFA	Ghana	Dimensional Change Card Sort (DCCS)^#^ task to measure EF	Positive findings - higher levels of DHA were associated with better EFs
Jumbe et al. (59)	Observational	analysis whole-blood FA profile in 2–6 years old	EFA and LC-PUFA	Tanzania	Dimensional Change Card Sort (DCCS)^#^ task to measure EF	Positive findings - whole blood levels of LA correlated with EF; Negative findings - whole blood levels of ALA and nervonic acid were inversely associated with EF.

RCT, randomized control trial; ALA, alpha-linolenic acid; LA, linoleic acid; EPA, eicosapentaenoic acid; DHA, docosahexaenoic acid; ARA, arachidonic acid; LCPUFA, long-chain poly-unsaturated fatty acids; WISC, Wechsler Intelligence Scale for Children; EF, executive functions; WM, working memory; NIRS, near infrared spectroscopy; DCS, diffuse correlation spectroscopy; DCCS, Dimensional Change Card Sort task; FA, fatty acids. All studies included were focusing on healthy subjects, as one of the exclusion criteria of this review was presence of physical, neurological, or psychological disorder, suffering from malnourishment or growth faltering. ^#^ indicates assessment of the subjects.

### 3.1 Lipid supplementation during pregnancy

Table 1 summarizes the main findings for fatty acid supplementation during pregnancy, with details on the study design, lipid intervention or assessment, age of participants, neural, cognitive, and/or behavioral assessment. Six studies of 12 studies showed positive effects of prenatal FAs (both EFA and LC-PUFA) supplementation on cognition (39–42, 44, 51). For example, after supplementation of 400 mg of DHA from 18 to 22 weeks of pregnancy until delivery, children of the supplemented mothers showed increased sustained attention at 5 years of age. Improved motor development was observed in children of mothers who received supplements containing LA, ALA, plus other micronutrients and vitamins from early pregnancy (< 21 weeks) until 6 months postpartum at 12 months of age, but not later at 18 months (45). A supplementation of omega-3 and omega-6 combined with micronutrients plus nutrition education to mothers resulted in an increase of motor, cognitive and language development at 2 years of age as well as social-emotional function at 32 months of age (44). With the same aforementioned prenatal supplementation of 400 mg DHA, decreased behavioral problems in children in preschool age, but no effects in other neurodevelopmental dimensions (cognitive, motor, and social-emotional) were observed between 4 and 6 years of age (40). An intervention in Bangladesh supplemented mothers with LA and ALA from 20 weeks of pregnancy until 6 months postpartum, resulting in improved motor performance and receptive language of children at 18 months of age (39) and at 24 months, these children performed much better in expressive language than those in the other supplementation groups using the same assessment tool. In a study of expecting Mexican women, Parra-Cabrera et al. (41) evaluated maternal diets during the third trimester using a Food frequency questionnaire (FFQ). The authors also assessed the blood levels of fatty acid of omega-3 and omega-6. Maternal diets high in ARA were associated with short latency Brainstem Auditory Evoked Potentials (BAEP), a measure of sensory motor integration, in infants at 18 months. However, DHA did not have the same association on BAEP. The remaining six of 12 studies showed no effect of prenatal FAs supplementation on neurodevelopment (43, 46–50). In an Indian study (46) daily DHA (400 mg) supplements administered to mothers from 20 weeks of pregnancy until 6 months postpartum did not impact infant neurodevelopment at 12 months of age. In a study in the Seychelles, no association was identified between maternal DHA and ARA status at 28 weeks of gestation with language, cognition, academic attainment, and executive function in children at 7 years (47). In Bangladesh, mental and psychomotor development at 18 months was not influenced by maternal supplementation with fish oil containing 1.2 g DHA and 1.8 g eicosapentaenoic acid (EPA) from 25 weeks of pregnancy until delivery, when compared to soy-oil which contained 2.25 g LA and 0.27 g ALA (48). Similar results were observed in a study conducted in Mexico, that used daily DHA from 18 to 22 weeks of gestation until delivery (49). Further in Mexico, supplementation of DHA from 18 to 22 weeks of gestation until delivery did not influence BAEP in infants at 1 or 3 months or visual-evoked potentials at 3 or 6 months (50). Finally, a long-term follow-up of the ALA and LA pre- and post-natal supplementation study resulted in an absence of impact on the autonomic nervous system development assessed using the emotion go/no-go and Racer Simon tasks (43).

In summary, of the six positive studies identified, four contained interventions with EFA, two with LC-PUFA (DHA) and one extrapolated the association of LC-PUFA (ARA) consumption with outcomes relevant to neurodevelopment. On the other hand, of the six studies showing no impact, four had interventions with LC-PUFA and only two with EFA, which investigated a very long-term follow-up at 9–11 years. Thus, most of the studies that offered prenatal supplements containing both ALA and LA had positive effects on child cognition (motor, language, and socioemotional development). Most of the studies that offered DHA either alone or in combination with other EFAs (ARA and EPA) as prenatal supplements, did not show any significant effect in infants except in increased sustained attention at 5 years old (51). For the study of Tofail et al. (48), it is important to note that these authors compared the impact of fish-oil, containing LC-PUFA DHA and ARA, to soy-oil, containing EFA, ALA and LA; rendering potentially more difficult to dissect the beneficial effect of each intervention as the “control” of the other arm was containing other types of fatty acids. Interestingly, the only study investigating a physiological marker, Brainstem Auditory Evoked Potential, reported a positive association was with maternal dietary intake of ARA (41). The outcome of these studies suggests that in low SES areas, maternal supplementation with EFAs was more systematically associated with a benefit for infant neurodevelopment compared to supplementation with LC-PUFA.

### 3.2 Lipid supplementation from infancy to 24 months

Six of 11 studies showed positive effects of lipids on neurodevelopmental outcomes in infants and children up to 24 months of age (39, 40, 42, 44, 52, 53). Table 2 summarizes the main findings in this age group, with details on the study design, lipid intervention or assessment, age of participants, neural, cognitive, and/or behavioral assessment. Unay et al. (52) reported that infants fed with a 500 mg DHA supplemented formula for the first 16 weeks of their lives, as well as exclusively breastfed infants, had faster neural response (BAEP) maturation after supplementation than those fed with standard formula. The second study measured cerebral blood flow in toddlers between the ages of 15 months and 2 years after supplementation with plant polyphenols and omega-3 fatty acids (255 mg DHA and 171 mg EPA), micronutrients and protein, in a fortified blended food for 23 weeks, compared to a traditional rice breakfast (53). Young children in the polyphenol plus omega-3 group increased cerebral blood flow and had higher scores on the working memory task than the control. In terms of the neurobehavioral outcomes, three studies showed positive effects. A study conducted in Ghana assessed the effects of 1 year supplementation with LA (4.6 mg) and ALA (0.6 mg) in infants (6–12 months old) and showed improved motor development at 12 and 24 months but not at 18 months, pointing to beneficial effects (42, 44); but no effect on the very-long term in a follow-up at 9–11 years of age (43). Another study also conducted in Ghana showed that the same lipid supplementation (4.6 mg LA and 0.6 mg ALA) from 6 to 12 months decreased behavioral problems in children at 18 months but did not affect cognition or fine motor function between 4 and 6 years of age (40). In a study conducted in Bangladesh, higher motor development scores as well as receptive language scores were reported in the same lipid supplemented group (4.6 mg LA and 0.6 mg ALA) from > 20 weeks of pregnancy until 6 months postpartum at 18 months (39), with a significant effect on expressive language reported at 24 months.

Noteworthy, five studies showed that EFAs or LC-PUFAs supplementation did not result in significant differences (42, 43, 54–56). Specifically in a study conducted in Malawi, the lipid supplementation with LA (4.6 mg) and ALA (0.6 mg) to children aged 6–18 months did not affect motor language, socio-emotional scores, or executive function skills (42). Similarly, mental development when assessed in 18 months olds was not impacted by a 12 months supplementation with either 3.6 g LA and 0.4 g ALA or 7.3 g LA and 0.8 g ALA (54). After 6 months fish-oil supplementation containing 200 mg DHA and 300 mg EPA, increased plasma n-3 fatty acid status was identified but there were no effects on attention and planning (55). Similarly, no association was identified between maternal essential fatty acid (DHA) status assessed at 2 months postpartum and visual acuity of infants at 2 months of age (56).

In summary, of the six positive studies, four had interventions containing EFA and two LC-PUFA, while of the five negative studies, three had interventions containing EFA and two LC-PUFA, so no definitive trend was observed. We can only extrapolate that the discrepancies from these lipid-based interventions might be due to study design differences or timing of supplementation. Interestingly, the two studies investigating BAEP and cerebral blood flow reported a positive impact of LC-PUFA (DHA) supplementation, which could indicate that these physiological markers might be important outcomes to measure the beneficial effects of nutrition in that population.

### 3.3 Lipid supplementation in children aged 2 years and above

Four studies out of five showed positive effects of lipids on cognition from 2 years and above (53, 57–59). Table 3 summarizes the main findings, with details on the study design, lipid intervention or assessment, age of participants, neural, cognitive, and/or behavioral assessment. In a South African study, improvements in verbal learning ability and memory were observed in children who received a fish-flour spread rich in EFA and LC-PUFA (335.02 mg ALA, 82.16 mg EPA, 475.44 mg DHA, 1567.36 mg LA and 23.25 mg ARA) for 6 months, compared to the control group (58). Another study conducted in Guinea Bissau used supplementation with high omega-3 LC-PUFA containing supplements (255 mg DHA and 171 mg EPA) in children between 2 and 4 years (53). This study showed increased cerebral blood flow and improved working memory in children who received the intervention (53). However, this outcome was not observed in children older than 4 years. In an observational study, children who had high plasma DHA levels (DHA > 4.0%) performed better on executive function tasks than those with the lower DHA levels (DHA < 2.0%) (57). Finally, in a study conducted in Tanzania, LA whole blood level was positively associated with executive functions, while ALA whole blood levels and nervonic acid showed an inverse association (59).

Only one study failed to report beneficial effect of EFA supplementation in children over 2 years of age, The Indian CHAMPION study supplemented children 6–10 years with 900 mg ALA plus 100 mg DHA or 140 mg ALA but had no effect on cognitive performance (i.e., short term memory, Fluid Reasoning, Cognitive Speediness and Overall Cognition) (60).

With a limited number of studies published in children 2–12 years of age, it is quite difficult to estimate the effects of lipid supplementation in this age group. Of the four studies showing a positive effect, two were interventions and two observational studies. One used an intervention with both EFA and LC-PUFA, two with either intervention or association with LC-PUFA and one had an association with EFA plasma levels. Thus, there might be slightly more evidence supporting a beneficial effect of LC-PUFA compared to EFA in that age-range. The only study investigating a physiological marker reported a beneficial effect of DHA on cerebral blood flow, again supporting the adequacy and sensitivity of physiological markers to identify beneficial effect of nutrition in early life. Despite the promising results in these limited studies, this age-range requires further investigation on the potential neurodevelopmental benefits of lipids consumption, especially in LMICs.

## 4 Discussion

The aim of this review was to provide a summary of the current literature assessing the impact of FAs on neurodevelopment in children living in LMIC. Our analysis included both intervention and observational studies conducted in Asia, Central America, and African countries that assessed the effects of pre- and postnatal supplements on neural, cognitive, and motor development of children up the age of 12 (all studies included in Supplementary Table 1). The strongest evidence identified in this narrative review is the beneficial impact of maternal intervention compared to intervention in infants or toddlers. Most fatty acids investigated in the RCTs were individual or mixed fatty acids including DHA, ARA, LA, and ALA. No specific effects of individual FA on any of the neurodevelopmental variables measured were consistently identified across the ages investigated. Studies that used ALA and LA supplementation during pregnancy showed a beneficial effect on postnatal cognitive and neurodevelopmental functions. On the contrary, in studies investigating intervention or observation in infants and toddlers, there was a lack of clear direction of the findings. Furthermore, in older children’s studies, the number of studies was limited and does not allow to reach any strong conclusion. At this stage, it is quite difficult to formulate generalized suggestions on the best or optimal lipid species (and associated lipid sources) that have the greatest impact on neurodevelopment and cognition throughout development. Based on the current evidence, supplements containing ALA (omega-3) and LA (omega-6) during pregnancy seem to result in the best outcomes and could be target of action aiming at improving diet in LMIC, which could be linked to their role as precursor for synthesis of ARA, DHA and EPA.

This review highlights that omega-3 and omega-6 FA are nutrients that could benefit infants neurodevelopment in LMIC, in particular as supplement to maternal diet. When it comes to LMIC, breast milk is the most affordable source of fatty acids as they are produced naturally and is the ideal source of nutrition throughout infancy if mothers are healthy and well-nourished. Numerous studies confirm that breastfeeding has a positive effect on long-term neurodevelopmental outcomes, cognitive functioning, and educational outcomes (10). More specifically, the major LC-PUFA in human milk are dihomo-gamma-linolenic acid, ARA (Omega-6), EPA and DHA (Omega-3), with worldwide mean levels of DHA and ARA in breast milk being 0.37% and 0.55% of total fatty acids, respectively (61, 62). According to the UNICEF guidelines, babies born and raised in LMIC are much more likely to be breastfed, up to the age of 2 years, especially due to the limited financial resources. Quoting Shahida Azfar, UNICEF’s Deputy Executive Director, “Breastfeeding is the best gift a mother, rich or poor, can give her child,” as there are no financial limitations (63). It is therefore essential for the mothers living in LMICs to have a diet rich in fatty acids, in order to build up optimal stores of EFAs in their milk to support healthy infants’ neurodevelopment (61). The Food and Agriculture Organization (FAO)/WHO has highlighted benefits of fish consumption during pregnancy for this reason. The official recommendation is to have regular consumption of fish or to use fish oil supplements in women even before pregnancy to build up the supply LC-PUFAs, in order to enhance the DHA concentration in milk during breastfeeding (64). A focus on the quality and presence of specific healthy fatty acids in the maternal diet is very important because it sets the base of neurodevelopment in the fetus and continues to nourish development throughout breastfeeding. Another important aspect to be considered for FA supplementation of omega-3 and omega-6 is the ratio between these two. Indeed, while BM contain roughly a 1:1 ratio, nutritional solution existing have more or less deviated from such optimal ratio. This point has been raised already since decades (36, 65). Thus any implementation of nutritional solution targeting LMIC would benefit from integrating such concern. Based on this information, a great step toward increasing EFA content in breast milk would be to provide affordable sources of high-quality fatty acids for maternal diets in LMICs. This in turn would have the potential to improve infant and child development with long term benefits on neurodevelopmental outcomes.

Only about 4% of children in LMICs are not breastfed. In these cases, infant formula can be used to supply the required nutrients and fatty acids to support neurodevelopment. Low breastfeeding rates may have financial implications for LMICs in the form of increased healthcare costs and reduced economic activity. In those cases of inadequate nutrition, fortification and provision of affordable formula as supplement to the diet of toddlers beyond 6 months of age could provide an adequate quantity of essential fatty acids (omega- 3 and omega-6), comparable to levels taken by infants continued to be breast-fed. The global standards suggested by FAO/WHO recommend that if DHA is added to a formula, then ARA contents should be at least at the same concentration level. Vegetable oils like corn, soy, safflower, olive, sunflower, soy, canola and linseed oils are often added to formula as a source of LA and ALA. Sunflower oil having the highest amounts of omega-6 PUFAs (65.70/100 g of total fat) and rapeseed oil having the highest amounts of omega-3 PUFAs (11.10/100 g of total fat) represent candidates for alternative solutions. However, it has been shown that these precursors of omega-3 and omega-6 fatty acids are not sufficiently synthesized from consumed precursors during early development to elicit beneficial effects, so it might remain necessary to include DHA and ARA to support healthy neurodevelopment (66, 67). Alternatively, fish and potentially algal oils, have been shown to also serve as affordable sources of LCPUFAs used in infant formula to support neurodevelopment in children who do not get the opportunity to be breastfed.

Given the relative high cost of fortified complementary foods in LMICs, most children usually share food that other members of the family consume during weaning. Usually, these diets are characterized by high amounts of carbohydrates and low amount of fats, that lead to deficits in FAs intake fundamental for neurodevelopment (55, 68). Furthermore, dietary FAs sourced from plants, fish or algae tend to be relatively expensive for families with low income resulting in either their omission, small portions, or lower quality of the dishes and products consumed. This leads to an inadequate supply of the required levels of fatty acids, in both children and expectant mothers, with consequences for neurodevelopment. Both the quality and quantity of FAs consumed become a problem.

Today, a balanced and healthy diet containing sufficient amounts of all macronutrients including FAs, is unaffordable for most people in LMICs. A healthy diet on average costs 69% more than an unhealthy one, which may translate to about 30% of the total house income (69). This is alarming and causes extra financial strains on lower socio-economic status families. Consequently, there is an urgent and global need to provide affordable sources of FAs in optimal amounts in LMICs, to support healthy neurodevelopment, while decreasing the total household expenditure on food to about 10%–15% (69).

There is still an important gap in the development of affordable sources of these FAs, an extensive review of the option has been performed by Qin et al. (70). Currently, oily fish and plants (nuts and seeds) are the main sources of omega-3 and omega-6 FAs used as dietary and supplementation sources (71). The main source of omega-3 and omega-6 fatty acids currently used in nutrition is fish, principally salmon, mackerel and herring (72). Alternative to fish sources are mainly plant or algae sources. When taking a closer look at different and affordable plant sources of FAs, sunflower and peanut oil contain high levels of LA while flaxseeds and canola oils contain relatively high concentrations of ALA (73). Recent studies have shown that low-cost sources of PUFAs (DHA, EPA, ARA) could be obtained from algal sources like *Thraustochytrium, Schizochytrium, Crypthecodinium, Phaeodactylum, Monodus* and *Mortierella* species (23, 74). The cost of extraction and purification are key issues with algal source of EFA and increasing exploration of new algae species could benefit the field (75). In addition to leveraging sources, either plant or algal, rich in EFA, research has been progressing in leveraging genetic modification of these organisms to optimize production of EFA (76). Thus, between leveraging plant or algal sources of EFA, implementing bioengineering to increase yield of these sources, there is promising horizon for alternative sources of EFA typically found in fish or fish oil. There is however, still some hurdle before implementation of such sources, and these are mainly to improve the efficiency of algal yield and to ensure safety of the produced EFA (77).

When evaluating the evidence on developmental effects of fatty acids, about two thirds of the studies reviewed here reported positive effects of lipids dietary intake on cognition, or association between plasma lipids and cognition in infants and children. The remaining of these concluded that the fatty acids investigated did not affect cognitive outcomes measured at various age stages. In particular, the ratio of studies showing positive vs. negative effect was 6:6 for prenatal intervention, 6:5 for postnatal intervention before 2 years, and 4:1 for intervention beyond 2 years of age. The heterogeneity in the observed results may be due to different factors. The reason for inconsistent findings at younger ages may be due to difficulties in administering proper neurodevelopment assessment tools in younger populations. In older children, properly accounting of all the potential confounding factors can bias the results and lead to higher occurrence of type II error. The tool used to measure neurodevelopment can be critical on the interpretation of the effect of an intervention. Indeed, multiple factors can impact the outcome such as, if the tool was global or country adapted, if the assessment was of general milestones achievement or of specific function, if the infant/toddler/children was directly assessed or indirectly assessed by questionnaire to parents/caregivers. While the full examination of the validity/adequacy of tools used to assess neurodevelopment is beyond the scope of this review, we ensured that all studies included have used age-adapted tools to assess neurodevelopment (78). Thus, while there are encouraging results, more research should be performed, especially in the infants beyond 2 years of age to confirm the current limited literature. Another element to be considered specific to LMIC, is the potential high propensity of nutritional deficiencies in the studies reviewed. Interestingly only five studies have reported the rate of stunting or underweight or anemia, for the first two, they navigate around 20%–25%, on the other hand, anemia ratio was reported to be around 66% (53, 57–60). While other manuscripts have not specifically reported the incidence of such status, we can extrapolate that other studies have incidences close to the one reported. Hence most of the infant would be anemic and a significant proportion would be underweighted or stunted. The beneficial effect identified in our review is therefore probably more specific to population suffering from some form of malnutrition.

Regarding the intervention multiple factors should be considered as key to the heterogeneity of the results reported, these include the type of lipid composition used in the intervention, initiation and/or duration of the intervention. Furthermore, two important aspects that can impact extensively the biological importance of the intervention have been ignored in these studies: genetic variance in fatty acid metabolism and the use of multi-factorial intervention. The metabolism of fatty acids in humans is affected by gene variants of Fatty Acid Desaturases (FADS), which is responsible for encoding the key enzymes (D5D and D6D) needed for fatty acid synthesis (79). Noteworthy, conversion of ALA into DHA and EPA and of LA into ARA is done by FADS but with low efficiency. Specifically, only 8%–20% of a dose of ingested ALA is converted into EPA and 0.5%–9% into DHA (80, 81). Only three studies in this review measured whole blood fatty acid levels, a critical parameter that might have affected the results from lipid intervention studies (52, 55, 59). This is an obvious limitation in the design of any nutritional intervention. Therefore, when studying the association between fatty acids and cognitive development, it might be more useful to measure blood plasma and red blood cell FA levels, in addition to measuring FA intake. Sampling blood is a systemic way to factor in genetic variation, thereby avoiding or reducing any potential confounding effect, (52, 79). This last point combined with the complexity to ensure adherence to intervention in any clinical trial warrants particular attention in any future trial. We strongly recommend that red blood cells EFAs status is measured as an essential step in studies investigating lipids interventions in early life. One additional points regarding FADS, is that in mother breastfeeding, the single nucleotides polymorphisms in FADS have been associated with breast milk fatty acids, suggesting that for the association studies leveraging milk analyses, maternal genotyping should be integrated as a co-variate (82). One additional aspect that could lead to the complex pattern of results observed is the impact of a LC-PUFA intervention on PUFA endogenous synthesis. Gibson et al. (65) reported that diets containing high levels of PUFAs reduced the endogenous synthesis of DHA from ALA, thus a complex ballet of interactions are in place between dietary intake, endogenous synthesis, genetic background that still needs to be fully understood.

One important limitation of this review is the limitations of existing publications; indeed, despite our best efforts we only found 27 manuscripts that matched our research criteria. From these studies, it was difficult to draw clear conclusions, due to the diversity of: intervention type (pure FA intervention, mix of FA, with or without additional micronutrients), time windows of the intervention (both in term of age of the subject, duration of the intervention and age at assessment), the endpoints measured (going from very specific functional assessments to general questionnaires of milestones), type of the studies (RCT vs. associations), inclusion of physiological readouts (rarely assessed despite usually having lower variability and less chances of bias) and the unavailability of genetic information (which may impact FA synthesis and bioavailability of dietary FA). While clearly highlighting the limitations of our review, we think that the importance of the topic, especially in the LMIC population, is paramount and requires additional research. This review aims at summarizing the current knowledge and highlighting improvements that can be made in research in this field.

Finally, global guidance by WHO recommends the need for interventions that provide multiple ways to improve child development. This is because healthy neurodevelopment depends on multiple factors, internal and external, that interact non-linearly throughout development. It follows that when studying the effects of an intervention it is also important to consider the extent to which environmental and social aspects impact neurocognition, including living and sanitary conditions at home, the school and social environment, access to appropriate learning materials, variety in sensory or social inputs, and visual and intellectual stimulation. These factors are important since it has been established that FAs do form a greater proportion of brain’s volume, but that brain growth is also largely affected by environmental factors (83–85). Only three studies identified in this review considered environmental stimulation as covariates, with varying results making it impossible to estimate the contribution of external factors on the relationship between lipid supplementation and cognitive functioning (48, 49, 51). In summary, when studying cognitive development in children coming from low SES, it is crucial to consider the contribution of environmental factors, in addition to nutrition, to assess how food and environment shape healthy neurodevelopment.

## 5 Conclusion

The evidence gathered showed differential effects on neurodevelopment, with most focus on the first year of life. Among all the supplements employed, sources of omega-3 and omega-6, in particular ALA and LA, showed the most consistent benefits in LMIC. However, the high variability in the type of FA, dosage, treatment duration and means of intervention, as well as the methods used to assess neurodevelopment did not allow for a unified conclusion on the linked benefits for healthy growth and brain development. Nevertheless, this work highlights the urgent need to adopt a unified approach when assessing lipid consumption in LMIC during development and provides a list of recommendations for future studies. These include: (i) to consider the cost of the FA-based supplements, in light of the country’s gross domestic product, food expenditure, and family income; (ii) to propose alternative and more affordable solutions, that respect both the quantity and the quality of FA sources in LMIC countries; (iii) when assessing benefits of lipid supplementation, and to take into the account potential genetic influences, complement intervention compliance or measures of dietary intake with direct outcome measures such as red blood cells fatty acids content when possible; (iv) to evaluate alternate sources of FA, such as algal sources; and finally; (v) when assessing neurodevelopment, cognitive functioning, motor skills, and healthy growth it is important to account for additional confounding factors, such as family income, stimulation from the environment, and the quality of school and home environment. Two actionable conclusions that can be derived from this review include: i. the recommendation of LA and ALA intake during gestation, as this was the one intervention associated almost systematically with improvement of neurodevelopment and ii. Further develop production for FA, especially EFA, leveraging development of more affordable vegetal or algal sources. Generally, it would help to build consensus and guidance for intervention studies on fatty acids and neurocognition in infants and children by providing guidance on protocols, food/supplement type, dosage, ages and appropriate tests to be employed to increase the reliability and reproducibility of interventions in this domain.
