# Supplementary material for: The importance of lipids for neurodevelopment in low and middle income countries
Source: Front Nutr. 2025 Jun 24;12:1488647. doi: 10.3389/fnut.2025.1488647 (PMC12234318; doi:10.3389/fnut.2025.1488647)
Supplement: Supplementary file 1 [file Table_1.docx]

## **Title**: Importance of lipids for neurodevelopment in low- and middle-income countries

## **Authors: Pervy Okai-Mensah; Diandra Brkic; & Jonas Hauser**

# **Supplementary table 1** Studies included in the review

| Authors | Type of Study | Fatty Acid Intervention / Assessment | Type of lipids in intervention or analyses | COUNTRY | Neurodevelopmental Assessment Tools | Results |
| --- | --- | --- | --- | --- | --- | --- |
| INTERVENTIONS | | | | | | |
| Khandelwal et al. (2020) | RTC with Intervention | 400 mg/d algal DHA from at most from 20 weeks pregnancy till 6 m postpartum | LC-PUFA | India | Development Assessment Scale for Indian Infants to assess neurodevelopment^#^ | Null findings - No effect on neurodevelopment at 12 m of age |
| Matias et al.  (2017) | RTC with Intervention | LA (4.59g) & ALA (0.59g) to mothers > 20 weeks pregnancy – 6 m. postpartum  LA (4.46g) & ALA (0.58g) for infants 6 – 24 m. (including multi vitamins and multi minerals) | EFA | Bangladesh | Developmental Milestones Checklist II*; Macarthur-Bates Communicative Development Inventory for expressive and receptive language*. | Positive findings - Improved motor performance and receptive language at 18 m of age but not in expressive language. Later at 24 m, lipid supplemented children performed much better in expressive language. |
| Ramakrishnan et al. (2015) | RTC with Intervention | 400 mg/day of DHA starting from 18 to 22 weeks of gestation through delivery | LC-PUFA | Mexico | Bayley Scales of Infant Development-II (BSID II)^#^ | Null findings - No effect on mental & psychomotor development at 18 m. |
| Ramakrishnan et al. (2016) | RTC with Intervention | 400 mg of DHA starting from 18 to 22 week of pregnancy until delivery. | LC-PUFA | Mexico | Conners’ Kiddie Continuous Performance Test for attention^#^ | Positive findings - Increased sustained attention in children at 5 years. |
| Stein et al.  (2012) | RTC with Intervention | 400 mg of DHA starting from 18 to 22 week of pregnancy until delivery. | LC-PUFA | Mexico | Sierra wave instrument (Cadwell laboratories) for auditory & visual evoked potentials^#^ | Null findings - No effect on brainstem auditory-evoked responses at 1 and 3 m or visual-evoked potentials at 3 and 6 m. |
| Tofail et al.  (2006) | RTC with Intervention | Fish oil, DHA (1.2g), EPA (1.8g) in intervention and soy-oil containing 2.25g LA and 0.27g ALA  > 25 weeks of pregnancy till delivery | LC-PUFA | Bangladesh | Bayley Scales of Infant Development-II (BSID II)^#^ | Null findings - No effect on mental & psychomotor development at 18 m. |
| Prado et al (2023) | RCT with intervention | Lipid based nutrient supplement containing LA (4.5 g or ALA 0.6g) from <20 weeks of pregnancy until 18 mo | EFA | Ghana | Strength and difficulties questionnaire (SDQ)*, Brief Problem Monitor – Parent (BPM-P)*, Mood and Feelings Questionnaire (MFQ)*, Screen for Child Anxiety-Related Emotional Disorders (SCARED)*, Early Adolescent Temperament Questionnaire (EATQ-P)*, Children’s Emotion Management Scales (CEMS)* | Null findings - No impact of intervention on social-emotional development at 9-11 years old. |
| Parra-Cabrera et al. (2008) | Observational | Evaluation of maternal dietary intake for PUFA, with specific focus on DHA and ARA | LC-PUFA | Mexico | Brainstem auditory evoked potential (BAEP)^#^ | Positive findings - Higher BAEP were associated with ARA, but not DHA in infants from 3 to 12 m. |
| Strain et al.  (2020) | Observational | Measurement of maternal EFA and PUFA status, but reported only DHA and ARA or total omega-3 and total omega-6 at 28 weeks gestation | LC-PUFA | Seychelles | Clinical Evaluation of Language Fundamentals^#^; Kaufman Brief Intelligence Test^#^; Woodcock-Johnson Test of Achievement–III^#^; Boston Naming Test ^#^and Trailmaking^#^ for EF. | Null findings - No association between maternal DHA and ARA status with language, cognition, scholastic achievement and executive function at age 7 years |
| Ocansey et al. (2019) | RCT with intervention | LA (4.59g) & ALA (0.59g) to mothers >20 weeks – 6 mo postpartum.  LA (4.46g) & ALA (0.58g) for infants 6-18 m (including multi vitamins and multi minerals) | EFA | Ghana | Developmental Neuropsychological assessment II – language^#^; Parental questionnaire for developmental status and milestones*; head-toe-knee-shoulders delayed gratification^#^ for EF; visual search tasks^#^, British Ability Scale II (BAS) ^#^ and Wechsler primary and preschool for Intelligence^#^ for IQ and visuospatial ability; Baddley memory task^#^ for WM; NIH toolbox^#^ for motor function; Strength and difficulties questionnaire* for socio-emotional questionnaire. | Positive findings - Decreased behavioural problems reported by caregivers at preschool age, especially for low stimulation households, but no effects on cognition or motor function at 4-6 y.o.(preschool age). |
| Phuka et al. (2012) | RCT with intervention | LA (3.6g) & ALA (0.4g), or LA (7.3g) & ALA (0.8g) to 6 mo old infants for 12 months (including multi vitamins and multi minerals) | EFA | Malawi | Griffiths’ Developmental Assessment Tool^#^ for cognitive development. | Null findings - No effect on cognitive development at 18 m. |
| Prado et al. (2016a) | RCT with intervention | LA (4.59g) & ALA (0.59g) to mothers > 20 weeks until 6 m. postpartum; LA (4.46g) & ALA (0.58g) to offspring 6-18 m (including multi vitamins and multi minerals) | EFA | Ghana | Kilifi Developmental Inventory^#^ for motor development; Macarthur-Bates Communicative Development Inventory* for language; Profile of social and emotional development*; A-not-B-task^#^ for EF | Positive findings - Improved motor development at 12 m., but not at 18 m.  Null findings - No difference in EF, motor, language or socio-emotional functions at 18 m. |
| Prado et al. (2016b) | RCT with intervention | LA (4.59g) & ALA (0.59g) to mothers > 20 weeks until 6 m. postpartum; LA (4.46g) & ALA (0.58g) to offspring 6-18 m (including multi vitamins and multi minerals) | EFA | Malawi | KDI^#^ for motor development; Macarthur-Bates Communicative Development Inventory* for language; Profile of social and emotional development*; A-not-B-task^#^ for EF | Null findings - Lipid based supplements taken from 6-18 m. had no effect on motor, language, EF, and socioemotional development at 18 m. |
| Unay et al. (2004) | RCT with intervention | DHA supplemented formula (0.5g/100g fat) to infants from 0 to 16 weeks (duration = 16 weeks). | LC-PUFA | Turkey | Brainstem auditory evoked potentials^#^ | Positive findings - Infants fed on human milk or a formula supplemented with LCPUFAs during the first 16 weeks showed more rapid brainstem auditory evoked potential maturation vs. standard formula-fed infants. |
| Van der Merwe et al. (2013) | RCT with intervention | Fish oil supplementation with DHA (200mg) & EPA (300mg) for 3-9m infants for 12 months | LC-PUFA | Gambia | Willatts’ 2-Step Infant Planning Test^#^ for EF | Null findings - Supplementation successfully increased plasma n-3 FA status in infants but had no effect on cognitive development post 6 m. of intake. |
| Aryee et al. (2024) | RCT with intervention | Small quantity lipid-based nutrient containing LA (4.5 g or ALA 0.6g) | EFA | Ghana | Autonomic nervous system assessed with Emotion Go/No-Go (EGEG) and RACER Simon^#^ | Null findings - No impact on impact on autonomic nervous system. |
| Imtiaz et al (2024) | RCT with intervention | Lipid based nutrient supplement containing LA (4.5 g or ALA 0.6g) with or without health education from <20 weeks of pregnancy until 23 mo of age | EFA | Pakistan | Caregiver-Reported Early Development Instrument (CREDI)* | Positive findings - Supplementation and education resulted in positive effect on cognition, motor and language development at 2 years and in those as well as social-emotional function at 32 months of age. |
| Krasevec et al. (2002) | Observational | Evaluation of breast milk and plasma 2 m. postpartum to assess FA profiles of total lipids (DHA) | EFA & LC-PUFA | Cuba | Teller acuity cards^#^ to measure visual acuity. | Null findings - No association between maternal DHA and infant visual acuity at 2 mo of age. |
| Dalton et al. (2009) | RCT with intervention | ALA (335.02mg), EPA (82.16mg), DHA (191.66mg), LA (1567.36mg), ARA (23.25mg) in a fish flour spread in children 7-9 y.o. for 6 m. | EFA & LC-PUFA | South Africa | Hopkins Verbal Learning Test^#^ for cognitive functioning, Reading and Spelling^#^ was also assessed. | Positive findings - Learning ability and memory improved after implementation containing LCPUFA |
| Muthayya et al. (2009) | RCT with intervention | ALA (930mg), DHA (100mg), or ALA (140mg) for 12 m. in children 6-10 y.o. (including multi vitamins and multi minerals) | EFA & LC-PUFA | India | Kaufman Assessment Battery for Children^#^, WISC^#^; Rey Auditory Verbal Learning test; and Neuropsychological assessment tool^#^ for short term memory, fluid reasoning, cognitive Speediness and overall cognition. | Null findings - FA supplementation made no difference among the groups on short term memory, fluid reasoning, cognitive speediness and overall cognition |
| Roberts et al. (2020) | RCT with intervention | DHA (255mg) & EPA (171mg) in children from 15 m - 7 y.o. for 23 weeks (including multi vitamins and multi minerals) | LC-PUFA | Guinea Bissau | Spin the pot task (variation)^#^ for WM; NIRS^#^ and DCS^#^ for cerebral flood. | Positive findings - Increased cerebral activity (blood flow) and better WM were observed in children < 4 y.o. but not in the 4 and above. |
| Adjepong et al. (2018) | Observational | analysis whole-blood FA profile in 2–6 y.o.s | EFA & LC-PUFA | Ghana | Dimensional Change Card Sort (DCCS)^#^ task to measure EF | Positive findings - Higher levels of DHA were associated with better EFs |
| Jumbe et al. (2016) | Observational | analysis whole-blood FA profile in 2-6 y.o. | EFA & LC-PUFA | Tanzania | Dimensional Change Card Sort (DCCS)^#^ task to measure EF | Positive findings - Whole blood levels of LA correlated with EF;  Negative findings - whole blood levels of ALA & nervonic acid were inversely associated with EF. |

Legend: RCT = Randomised Control Trial; ALA = alpha-linolenic acid; LA = linoleic acid; EPA = Eicosapentaenoic acid; DHA = docosahexaenoic acid; ARA = arachidonic acid; FA = fatty acids; yo= years old; m= months; KDI= Kilifi Developmental Inventory; K-CPT = Conners’ Kiddie Continuous Performance Test; EFs= Executive functions; BSID = Bayley Scales of Infant Development; BAEP=brainstem auditory evoked potential; BAS= British Ability Scale II; WM= working memory; NIRS = near infrared spectroscopy; DCS= diffuse correlation spectroscopy; LCPUFA= long-chain polyunsaturated fatty acids; WISC= Wechsler Intelligence Scale for Children; DCCS=Dimensional Change Card Sort task;
